# Strategies for the Viral Exploitation of Nuclear Pore Transport Pathways

**DOI:** 10.3390/v17020151

**Published:** 2025-01-23

**Authors:** Xin Zhang, Keesiang Lim, Yujia Qiu, Masaharu Hazawa, Richard W. Wong

**Affiliations:** 1 Division of Nano Life Science, Graduate School of Frontier Science Initiative, Kanazawa University, Kanazawa 920-1192, Japan; xinzx1995@stu.kanazawa-u.ac.jp (X.Z.); qyj@stu.kanazawa-u.ac.jp (Y.Q.); 2 WPI-Nano Life Science Institute, Kanazawa University, Kanazawa 920-1192, Japan; mhazawa@staff.kanazawa-u.ac.jp; 3 Cell-Bionomics Research Unit, Institute for Frontier Science Initiative, Kanazawa University, Kanazawa 920-1192, Japan

**Keywords:** spider cobweb, central plug, gene gating, super enhancer, nucleoporins, karyopherin, SARS-CoV-2, HIV, antiviral drug, nuclear transport inhibitor, HS-AFM

## Abstract

Viruses frequently exploit the host’s nucleocytoplasmic trafficking machinery to facilitate their replication and evade immune defenses. By encoding specialized proteins and other components, they strategically target host nuclear transport receptors (NTRs) and nucleoporins within the spiderweb-like inner channel of the nuclear pore complex (NPC), enabling efficient access to the host nucleus. This review explores the intricate mechanisms governing the nuclear import and export of viral components, with a focus on the interplay between viral factors and host determinants that are essential for these processes. Given the pivotal role of nucleocytoplasmic shuttling in the viral life cycle, we also examine therapeutic strategies aimed at disrupting the host’s nuclear transport pathways. This includes evaluating the efficacy of pharmacological inhibitors in impairing viral replication and assessing their potential as antiviral treatments. Furthermore, we emphasize the need for continued research to develop targeted therapies that leverage vulnerabilities in nucleocytoplasmic trafficking. Emerging high-resolution techniques, such as advanced imaging and computational modeling, are transforming our understanding of the dynamic interactions between viruses and the NPC. These cutting-edge tools are driving progress in identifying novel therapeutic opportunities and uncovering deeper insights into viral pathogenesis. This review highlights the importance of these advancements in paving the way for innovative antiviral strategies.

## 1. Introduction

Pathogenic viruses, a significant class of infectious agents, have been responsible for widespread morbidity and mortality, profoundly impacting global public health. Beyond their immediate effects on human health, viral outbreaks have led to severe socioeconomic consequences, disrupting economies, straining healthcare systems, and altering societal structures. These pathogens, ranging from influenza and HIV to emerging threats like SARS-CoV-2, underscore the critical need for ongoing research and preparedness to mitigate their far-reaching effects. Over the past century, humanity has faced two major pandemics: the 1918 Spanish Flu and the recent COVID-19 crisis. Despite remarkable advancements in science and technology, communicable diseases persist as major global challenges, with the development of effective antiviral therapies and vaccines remaining an arduous endeavor.

Throughout their lifecycle, many viruses strategically exploit the host’s transcriptional and translational machinery to facilitate replication while simultaneously evading immune defenses to ensure survival and propagation [1]. The nucleoplasm and cytoplasm of human cells are compartmentalized by the nuclear envelope (NE), a double lipid bilayer that houses nuclear pore complexes (NPCs) (reviewed in [2,3,4,5,6,7,8]). These highly specialized nano-gates regulate nucleocytoplasmic transport, a critical process for maintaining cellular homeostasis. NPCs play a pivotal role in facilitating the bidirectional exchange of macromolecules, such as RNA, proteins, and ribonucleoproteins, between the nucleus and cytoplasm. This transport is essential for a wide range of cellular functions, including cell division [4,9], metabolic regulation [10], gene expression and regulation [3], and the activation of innate immune responses [10,11]. By tightly controlling nucleocytoplasmic trafficking, NPCs serve as key regulators of cellular processes, making them a target for viral exploitation during infection.

Viruses depend extensively on hijacking the host’s nuclear transport machinery for replication, making these processes promising targets for antiviral therapies. Disrupting nuclear trafficking has the potential to inhibit critical viral activities, including genome transcription, protein synthesis, and assembly. This review investigates the strategies employed by viruses, with a particular focus on SARS-CoV-2 and human immunodeficiency virus (HIV), to exploit host nucleocytoplasmic trafficking. Emphasis is placed on viral components and their interactions with host factors, such as importins and nucleoporins (Nups). Additionally, we explore the therapeutic potential of targeting host nuclear transport mechanisms, offering insights into innovative approaches to combat viral infections.

## 2. The ABCs of NPCs

**Figure 1:**
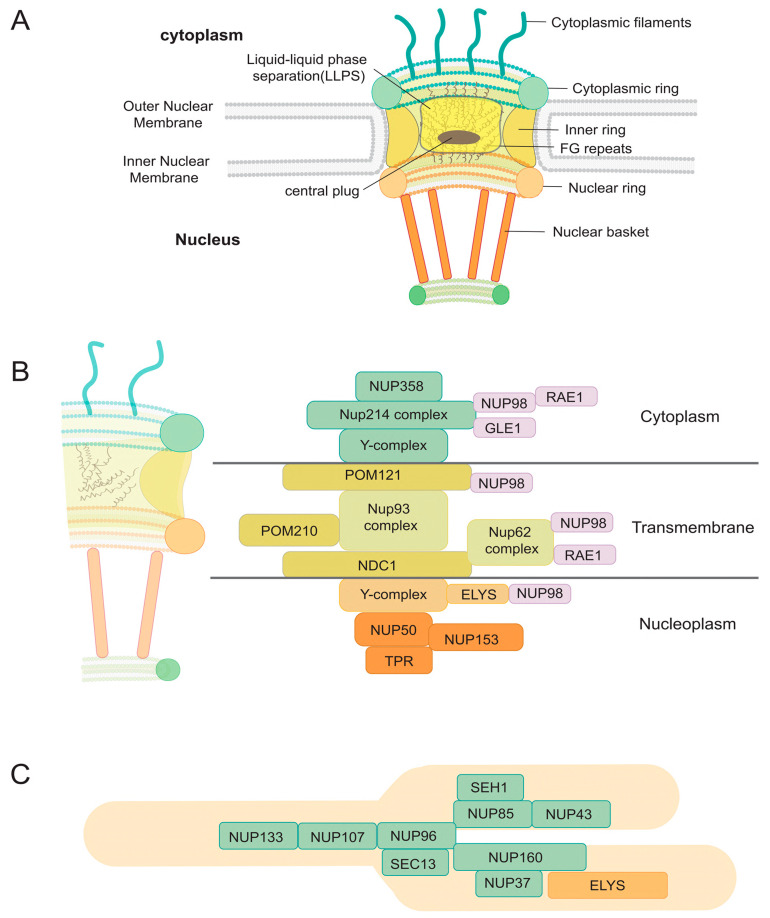
Schematic representation of the nuclear pore complex (NPC) and its role in nuclear import and export processes. The NPC is embedded in the nuclear envelope and consists of nucleoporins (Nups) organized into structural elements, including the inner ring, cytoplasmic and nuclear rings, cytoplasmic filaments, and the nuclear basket [35]. A circular scaffold, known as the luminal ring, surrounds the NPC within the lumen of the nuclear envelope, providing structural integrity. The central channel, lined with FG-Nups, features a dynamic spider cobweb-like network that facilitates selective transport while maintaining a permeability barrier. Additionally, a central plug structure, often visualized during transport processes, may represent transient cargo complexes or structural rearrangements, emphasizing the NPC’s functional versatility in nucleocytoplasmic trafficking. This schematic underscores the intricate architecture and regulatory mechanisms of the NPC in facilitating macromolecular transport. (**A**) Structural Overview of the NPC Core and Peripheral Components. The central core of the NPC consists of several structural components, including the Cytoplasmic Ring (CR), Nuclear Ring (NR), Luminal Ring (LR), and Inner Ring (IR). Peripheral elements, such as the Cytoplasmic Filaments (CF) and Nuclear Basket (NB), extend into the cytoplasm and nucleus, respectively. The IR encircles the NPC’s core channel, which is densely lined with FG-Nups that form a cohesive diffusion barrier, enabling selective macromolecular transport between the cytoplasm and nucleus. (**B**) Schematic Representation of Nucleoporin Complexes. A schematic diagram illustrates the spatial organization of nucleoporin complexes within the NPC, including those forming the central core and peripheral structures. This visualization highlights their structural roles in maintaining NPC integrity and their functional contributions to nucleocytoplasmic transport. (**C**) Detailed Diagram of the Y-Shaped Coat Nucleoporin Complex (CNC). The Y-shaped CNC is depicted in detail, showcasing its structural components. The stem region comprises NUP133, NUP107, NUP96, and SEC13, while the short arm includes SEH1, NUP85, and NUP43. The long arm features NUP37 and NUP160. Additionally, the asymmetric ELYS protein is localized on the nuclear side, where it plays a pivotal role in NPC assembly and anchoring to the nuclear envelope. Collectively, these structural and functional components contribute to the NPC’s ability to regulate selective nucleocytoplasmic transport, underscoring its architectural and functional complexity.

NPCs are large protein assemblies, with a molecular mass of approximately 50 MDa in yeast and 110 to 125 MDa in metazoans [12,13]. The outside diameter ranges from 80 to 120 nm, whereas the interior diameter is approximately 40 nm [14,15,16,17]. Each NPC consists of numerous copies (between 8 and 64) of around 34 distinct nuclear pore proteins, referred to as Nups (designated Nup followed by their estimated molecular weight), the majority of which are conserved across various animals (Figure 1). These Nups, found in quantities between 8 and 48, aggregate into several rings arranged along the NE [18,19,20,21]. The Y-shaped structure of the NPC is a hallmark architectural feature observed in its cytoplasmic and nucleoplasmic rings, which anchor the central transport channel to the nuclear envelope. These Y-shaped subcomplexes are composed primarily of scaffold nucleoporins, forming an intricate lattice that provides both mechanical stability and spatial organization to the NPC. The arms of the Y-shaped structures interact with the lipid bilayer of the nuclear envelope, ensuring the proper curvature and integration of the pore within the membrane. Meanwhile, their base serves as a docking site for other nucleoporins, facilitating the assembly and maintenance of the NPC’s selective permeability barrier. This configuration is critical for the bidirectional transport of macromolecules, as it establishes the structural foundation for the dynamic gating mechanisms mediated by nucleoporins with phenylalanine-glycine repeats or FG-Nups [14,15,16,17,22].

The central channel of the NPC is lined with FG-Nups. These repeats interact to form a cohesive meshwork that appears to phase-separate from the surrounding solution, functioning as a selective permeability barrier. This barrier regulates nucleocytoplasmic transport and is often described through models such as the “oily spaghetti” [23], “hydrogel [24]”, “polymer brush [25]”, “forest model [26]”, “reduction of dimensionality model [27]”, and “cobweb model [28]”, each attempting to explain the intricate, multilayered structure of FG-Nups. The “cobweb model” analogy is particularly apt, highlighting the dynamic, spiderweb-like arrangement of flexible FG-Nups that create a selective barrier for macromolecular transport [16,17].

This FG-Nup meshwork prevents the passive diffusion of macromolecules and complexes larger than approximately 30–40 kDa, such as proteins, RNAs, and viruses. To traverse the NPC, these larger macromolecules require active transport mediated by nuclear transport receptors or NTRs (importins, exportins, transportins, or karyopherins), which facilitate their passage through the selective barrier. This sophisticated system ensures efficient and regulated nucleocytoplasmic transport, balancing cellular needs with strict selectivity.

This comprises outer rings on the nuclear and cytoplasmic sides (nuclear ring [NR] and cytoplasmic ring [CR]) and an inner ring (IR) situated between them. Each ring typically comprises eight recurring parts. FG repeats of FG-Nups extend inward from these rings to create the core channel of the nuclear pore complex and engage with transport factors to facilitate selective nucleocytoplasmic transport [18,29].

Despite advances in understanding the conserved core architecture, outlying regions can vary greatly within and between species. A cage-shaped nuclear receptacle is an example. Despite its importance in mRNA monitoring and chromatin assembly, its architecture is unknown. Singh et al. used in-cell cryo-electron tomography and subtomogram analysis to study the nuclear basket and NPC structures in fungi (*S. cerevisiae*), mammals (*M. musculus*), and protozoa (*T. gondii*) [30]. Integrative structural modeling shows how a nuclear ring hub of Nups connects to basket-forming Mlp/Tpr proteins. The basket’s struts are Mlp/Tpr’s coiled-coil domains, while its distal densities are its unstructured termini. Distal densities may dock mRNA preparation before nucleocytoplasmic transit.

The central plug, or central transporter, within the NPC represents a foundational step in understanding NPC architecture. First described by Unwin and Milligan in 1982 through cryo-Electron Microscopy (cryo-EM) studies of Xenopus oocytes, the central plug was identified as a spheroidal structure approximately 35 nm in diameter located at the core of some NPCs [31]. Despite its early characterization, the composition and function of the central plug remain subjects of debate. It is generally accepted as a dynamic structure that appears under specific conditions. A 2020 study by Mohamed et al., utilizing high-speed atomic force microscopy (HS-AFM) [32], provided new insights, suggesting that the plug is at least partially composed of FG-Nups [33]. Their observations indicated that FG-Nups form transient “knots” within the pore’s core and that plugs in cancerous HCT116 cells exhibit greater conformational dynamics than those in non-cancerous colon cells. The origins of the central plug model stem from the limitations of early cryo-EM, which required immobilized samples, leading to a static representation of the NPC. As imaging technologies have advanced, so has our understanding of NPC dynamics, refining the plug model to better reflect its functional complexity. Emerging live-cell data promise to uncover the elusive identity and role of the central plug, potentially resolving a longstanding question in cell biology [34].

**Figure 2:**
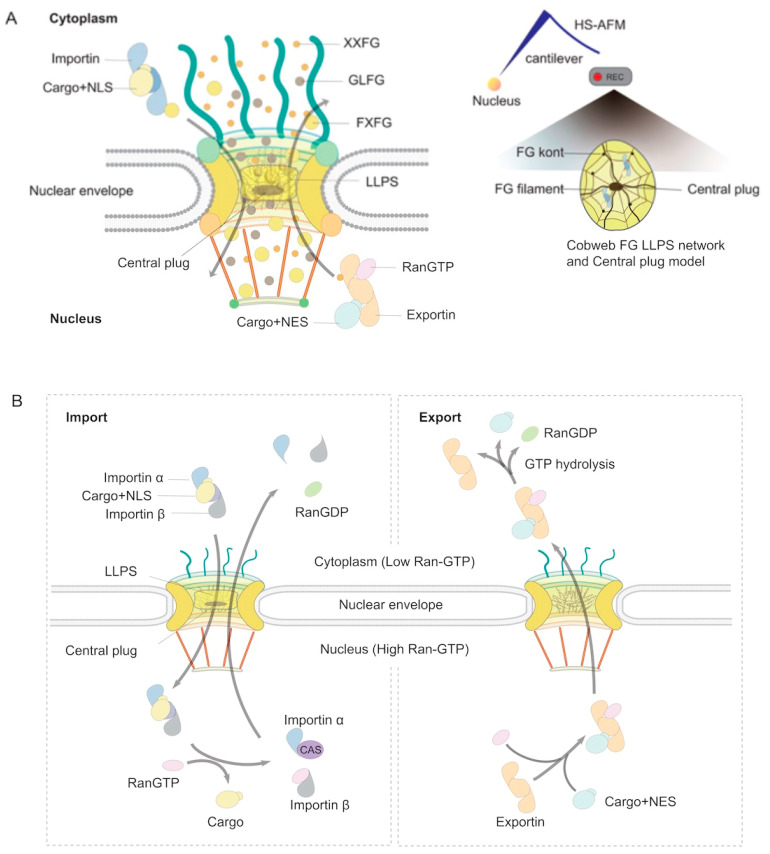
Principles of nucleocytoplasmic transport and the model of protein nuclear import and export. (**A**) FG Filaments, Central Plug, and Directional Transport in the NPC. The NPC’s selective transport is governed by the spatial distribution of FG-repeat types, including XXFG (small spheres), GLFG (medium spheres), and FXFG (large spheres), which establish distinct concentration gradients within the NPC channel. FG filaments, visualized by HS-AFM as a spider cobweb-like central plug (CP), form a cohesive and dynamic network critical for maintaining selective permeability. In the cytoplasm, NLS-containing cargoes bind to importins, inducing conformational changes in their HEAT repeats, creating high-affinity binding pockets for FXFG repeats. This interaction, along with the FG gradient, propels the importin-cargo complex directionally toward the nucleus. Within the nucleus, RanGTP binding to importins reduces the size of these pockets and shifts their specificity toward XXFG repeats, facilitating cargo release and preparing for export. Exportins utilize similar FG gradients to mediate NES-dependent nuclear export [54]. The concentration gradients of FG-repeat types provide directional traction forces, ensuring unidirectional and efficient transport. Peripheral structures, such as cytoplasmic filaments and the nuclear basket, play crucial roles in anchoring transport receptors and enhancing the dynamics and efficiency of nucleocytoplasmic transport. (**B**) Mechanisms of Nuclear Translocation. Nuclear translocation involves the bidirectional movement of specific cargoes mediated by NLSs and NESs. In the cytoplasm, NLS-bearing cargoes bind to importins and traverse the NPC. Inside the nucleus, RanGTP induces conformational changes in the importin-cargo complex, triggering cargo release. Conversely, nuclear export begins with the formation of a trimeric complex comprising an exportin, NES-bearing cargo, and RanGTP. This complex translocates through the NPC, and RanGTP hydrolysis in the cytoplasm triggers its disassembly. Additionally, specific RNA species require protein adaptors for NPC-mediated transport, ensuring precise regulation of RNA export pathways. The interplay between these mechanisms and the NPC’s structural features ensures highly regulated nucleocytoplasmic transport.

In contrast to Cryo-EM [36,37,38,39], Nuclear Magnetic Resonance (NMR) or conventional microscopy techniques for visualizing and analyzing NPCs, HS-AFM is a state-of-the-art imaging technique that allows for the direct visualization of individual biomolecules in real time, offering unprecedented insights into their dynamic behavior [32,40,41]. HS-AFM provides high temporal resolution, enabling the capture of molecular movements and interactions as they unfold. This technology is particularly suited for investigating nanoscale biomolecular processes, including protein conformational changes, molecular assembly, and ligand-receptor interactions. Its combined high spatial and temporal resolution has made HS-AFM an indispensable tool in structural biology, revealing intricate details of biomolecular mechanisms that are otherwise difficult to observe [42]. Our experience with HS-AFM demonstrates its versatility and power, as seen in studies of human NPCs [28,33], DNA-protein interactions [43], viral proteins, viral protein-antibody interactions [44,45], and viral protein-organelle interactions [44,46,47,48]. These applications underscore the transformative potential of HS-AFM in advancing our understanding of complex biological systems [42]. HS-AFM has also revolutionized the structural and functional analysis of human NPCs, providing unprecedented nanometer-scale resolution and real-time visualization [28,33,49,50]. HS-AFM has captured the adaptive nature of these cobweb-like structures, revealing how NPCs rearrange during active transport, chromatin reorganization, and stress responses (Figure 2). These observations underscore the NPC’s role not only as a static gatekeeper but also as a dynamic scaffold, facilitating interactions with genome regulators and responding to cellular demands. By providing real-time insights into the cobweb-like architecture and function of NPCs, HS-AFM has deepened our understanding of their critical roles in maintaining nuclear-cytoplasmic homeostasis and genome stability [42].

Nuclear transport receptors (NTRs) regulate the import and export of macromolecular cargoes via the small Ras-like GTPase Ran, which cycles between GDP- and GTP-bound states [51]. In the nucleus, the guanine exchange factor RCC1 converts Ran-GDP to Ran-GTP, while GTP hydrolysis, catalyzed by Ran GTPase-activating protein 1 (RanGAP1) and stimulated by RanBP2/Nup358 on the cytoplasmic filaments, converts Ran-GTP back to Ran-GDP. This compartmentalized activity creates a ∼200-fold Ran-GTP gradient favoring the nucleus. Nuclear transport factor 2 (NTF2) ferries Ran-GDP into the nucleus, ensuring a high Ran concentration in the nucleoplasm, where GTP hydrolysis powers Ran-dependent import and export [52,53] (Figure 2).

Proteins containing nuclear localization signals (NLSs) bind importin-α (also known as karyopherin-α), which interacts with importin-β (also known as karyopherin-β). Importin-β docks at FG-Nups to traverse the NPC. Inside the nucleus, Ran-GTP disassembles the importin-cargo complex, freeing cargo and recycling importin-β back to the cytoplasm, while importin-α is exported by CAS/exportin2. Noncanonical NLSs, such as proline-tyrosine motifs in hnRNPs, are recognized by transportin-1. Importin-β can also directly transport some cargoes, including ribosomal proteins and HIV proteins such as Rev and Tat.

Nuclear export involves leucine-rich nuclear export signals (NESs) recognized by exportin CRM1 (XPO1), which forms a trimeric complex with Ran-GTP and cargo. CRM1 binds FG-Nups to translocate through the NPC. GTP hydrolysis in the cytoplasm dissociates the complex, releasing cargo while CRM1 recycles to the nucleus. For noncoding RNAs, CRM1 uses adapter proteins such as PHAX to bind cargoes. mRNA export, distinct from protein and noncoding RNA pathways, involves the NXF1/NXT1 heterodimer recruited by the TREX complex and serine/arginine-rich proteins during transcription and RNA processing. NXF1/NXT1 binds FG-Nups to move messenger ribonucleoprotein (mRNP) complexes through the NPC. Cytoplasmic Dbp5 and RAE1/Gle2 remodel mRNPs in an ATP-dependent manner, preventing nuclear re-entry. TREX2 interacts with NPC components such as TPR and Nup153 to further facilitate mRNA export, though its mechanisms remain incompletely understood. This finely tuned transport system ensures efficient and selective nucleocytoplasmic exchange critical for cellular function [11,54]. Recently, Mishra et al. employed coarse-grained molecular dynamics simulations to explore the mechanisms underlying karyopherins (Kaps) translocation through NPCs [55]. Their study focused on how the surface charge and hydrophobicity of Kaps influence transport rates. They discovered that the negative surface charge of Kaps is crucial for successful translocation, while the hydrophobicity of transport particles enhances their movement through the NPC. Notably, their findings highlighted the significance of the positive net charge of Nups, particularly Nup1, in facilitating Kap transport. This charge creates a gradient of increasing binding affinity for Kaps with FG-Nups, progressing from the cytoplasm to the nucleus. This gradient, previously proposed but now further supported, provides key insights into the directional and efficient translocation of Kaps through the NPC [55].

Over the past few decades, research has unveiled the multifaceted roles of nucleoporins, the key components of the NPC. Beyond their fundamental function in forming the NPC structure, nucleoporins are critical for a diverse array of cellular processes. We and others have shown that NPC facilitates selective nucleocytoplasmic transport, enabling the exchange of macromolecules such as proteins and RNA between the nucleus and cytoplasm. However, nucleoporins also contribute to processes independent of transport, including mitosis, where they regulate spindle assembly and chromosome segregation [4,56,57,58,59,60,61,62,63,64]. Additionally, they play pivotal roles in gene expression by influencing chromatin organization and transcriptional activity [65,66,67].

Gene gating is a process in which transcriptionally active genes are positioned adjacent to NPCs, facilitating the efficient maturation of nascent transcripts into export-ready mRNA by associating them with export factors. This concept was first proposed by Günter Blobel in 1985, offering a framework to understand the spatial coupling of transcription and mRNA export within the nucleus [68]. Hazawa et al. demonstrated that master transcription factors, such as TP63, establish super-enhancers (SEs) to drive core transcriptional networks in cancer cells [66]. However, the spatiotemporal regulation of SEs within the nucleus remains largely unexplored. Their study revealed that the NPC may tether SEs to the nuclear periphery, where RNA export rates are highest. Specifically, NUP153, a key component of the NPC, was found to anchor SEs to the pore, enhancing TP63 expression by maximizing mRNA export. This anchoring is facilitated by protein-protein interactions between the intrinsically disordered regions (IDRs) of NUP153 and the coactivator BRD4 [69]. Silencing NUP153 disrupts SE localization, displacing them from the nuclear periphery, which in turn reduces TP63 expression, impairs cellular growth, and promotes epidermal differentiation in squamous cell carcinoma. This study first highlights the critical role of NUP153 IDRs in regulating SE localization and provides new insights into the spatial and epigenomic layers of gene gating [66,70].

Nucleoporins are also essential for proper organismal development, as they mediate tissue-specific gene regulation and signaling pathways. Dysregulation of NPC components has been implicated in various diseases, including cancer, neurodegenerative disorders [71], and viral infections, underscoring their importance in maintaining cellular homeostasis and responding to pathological challenges [72,73,74].

Recently, Ikliptikawati et al. identified the genomic amplification of NUP107, a core component of the NPC, in glioblastoma (GBM), revealing its overexpression alongside MDM2, a critical E3 ligase responsible for p53 degradation [67]. The depletion of NUP107 in GBM cell lines suppresses growth by stabilizing p53 protein levels. Mechanistically, the study uncovered that NPCs act as a platform for p53 degradation by coupling nuclear export pathways with 26S proteasome tethering. As a keystone for NPC assembly, NUP107 is essential for maintaining NPC structural integrity. Loss of NUP107 disrupts NPC architecture, significantly reducing the proximity of 26S proteasomes to nuclear pores and impairing their role in p53 degradation. These findings underscore the dual roles of NPCs in transport surveillance and protein homeostasis, while offering new insights into mechanisms of p53 inactivation in GBM [3].

These insights highlight the NPC as not only a structural gateway but also a dynamic regulator of essential cellular functions [3,75,76,77,78,79,80,81,82,83].

## 3. Hijacking Mechanisms of Host Nuclear Transport Machinery by Viruses

Viruses frequently hijack critical NTRs, such as importin-α/β, Crm1, and Nxf1, as well as NPC components including Nup98, Rae1, Nup358, and Nup214, to facilitate the trafficking of their components between the cytoplasm and nucleus. The extent and mechanism by which host nuclear transport is exploited largely depend on the site of viral replication. By manipulating these essential pathways, viruses ensure the efficient delivery of their genetic material and proteins to appropriate subcellular locations, effectively undermining host cellular defenses and enabling replication. Cytoplasmic-replicating viruses often hijack nuclear transport to suppress interferon (IFN)-mediated antiviral responses, while nuclear-replicating viruses use these mechanisms for tasks such as importing viral genomes into the nucleus and exporting newly synthesized viral RNA to the cytoplasm. This review categorizes viral strategies based on replication sites: cytoplasm or nucleus.

### 3.1. Viruses That Replicate in the Cytoplasm

#### 3.1.1. β-Coronaviruses (SARS-CoV-2, SARS-CoV-1, and MERS-CoV)

Coronaviruses (CoVs), members of the Coronaviridae family, are enveloped viruses distinguished by their positive-sense single-stranded RNA genome. These viruses are classified into four genera: Alphacoronavirus (α-CoV), Betacoronavirus (β-CoV), Gammacoronavirus (γ-CoV), and Deltacoronavirus (δ-CoV) [84]. While most human coronaviruses (HCoVs) are associated with mild upper respiratory or gastrointestinal illnesses, such as the common cold (reviewed in [85]), zoonotic coronaviruses within the β-CoV genus have caused severe outbreaks of lower respiratory tract diseases with high mortality rates. Notable examples include the severe acute respiratory syndrome coronavirus (SARS-CoV) and the Middle East respiratory syndrome coronavirus (MERS-CoV), both of which emerged from animal reservoirs and caused significant global health concerns (reviewed in [84]). More recently, SARS-CoV-2, a highly transmissible Betacoronavirus, was first identified in Wuhan, China, in late 2019. This virus has been responsible for the most devastating pandemic of the 21st century, causing widespread morbidity, mortality, and unprecedented socioeconomic disruption [85]. The emergence of SARS-CoV-2 underscores the ongoing threat posed by zoonotic coronaviruses and highlights the critical need for preparedness and surveillance. Within cytosolic double-membrane vesicles (DMVs), all coronaviruses, including SARS-CoV-2, are capable of replicating [86,87]. Using HS-AFM, Lim et al. investigated the molecular properties of an S neutralizing antibody (S NAb) and its interaction with spike proteins [46]. At low density, the S NAb adopted a monomeric Y-shaped conformation, while at high density, it assembled into hexameric oligomers. The dynamic interaction between the S NAb and the spike protein at the receptor-binding domain (RBD) neither triggered RBD opening nor caused shedding of the S1 subunit. Notably, this interaction remained stable under endosomal pH conditions, suggesting a minimal risk of antibody-dependent enhancement (ADE). Additionally, the dynamic behavior of spike proteins on small extracellular vesicles (S sEVs) closely resembled that observed on SARS-CoV-2 virions. HS-AFM was shown to be a powerful tool for evaluating the sensitivity of variant S sEVs to S NAb binding. Collectively, Lim et al. introduced a nanoscopic assessment platform capable of providing detailed insights into the binding properties of S NAbs, advancing our understanding of antibody-spike protein interactions [46].

Through the use of cryo-electron microscopy and sub-tomogram averaging, Huang and his colleagues were able to successfully characterize the pores that are produced in DMVs. Based on the findings, it was determined that the pore is composed of twelve copies of both nsp3 and nsp4, which are organized in four concentric, stacked hexameric rings, giving it the appearance of a miniature NPC [88].

Protein-protein interaction (PPI) investigations indicated that SARS-CoV-2 proteins NSP9 [89], NSP15, and Orf6 interact with the host’s nuclear transport system [90]; however, only Orf6 has been verified to interfere with nucleocytoplasmic trafficking [91,92,93,94,95]. Orf6 interacts with Nup98 and Rae1, resulting in the mislocalization of these nucleoporins and the inhibition of nuclear import [93]. It furthermore binds importin-α1, inhibiting IRF3 nuclear translocation and decreasing type I IFN synthesis [95]. Moreover, Orf6 inhibits the nuclear import of STAT1/STAT2, hence obstructing the transcription of interferon-stimulated genes (ISGs) [92]. SARS-CoV-1 Orf6 disrupts IFN signaling through a multifaceted mechanism involving nuclear transport inhibition. It binds directly to importin-α1, a key nuclear transport adaptor, and simultaneously sequesters importin-β1 within the endoplasmic reticulum (ER)/Golgi membrane. This dual action effectively blocks the nuclear translocation of phosphorylated STAT1 (pY-STAT1), a critical transcription factor in the IFN signaling pathway. By preventing pY-STAT1 from reaching the nucleus, Orf6 inhibits the activation of interferon-stimulated genes (ISGs), thereby suppressing the host’s antiviral immune response. This strategy exemplifies how SARS-CoV-1 leverages host nuclear transport machinery to evade immune defenses [92,93,94,95]. SARS-CoV-2 Orf6 demonstrates more robust interactions with Nup98 and Rae1 compared to SARS-CoV-1 Orf6, which may account for the increased incidence of asymptomatic infections associated with SARS-CoV-2 [94]. Yoo and Mitchison similarly reported that SARS-CoV-2 ORF6 is 15 times more effective than SARS-CoV-1 ORF6 in obstructing nuclear transport [96]. Additionally, they suggested that the hydrophobic N-terminal domain facilitates the oligomerization of ORF6, allowing it to cross-link with Nup98 and Rae1 at the nuclear pore complex to inhibit nuclear transport [96].

**Figure 3:**
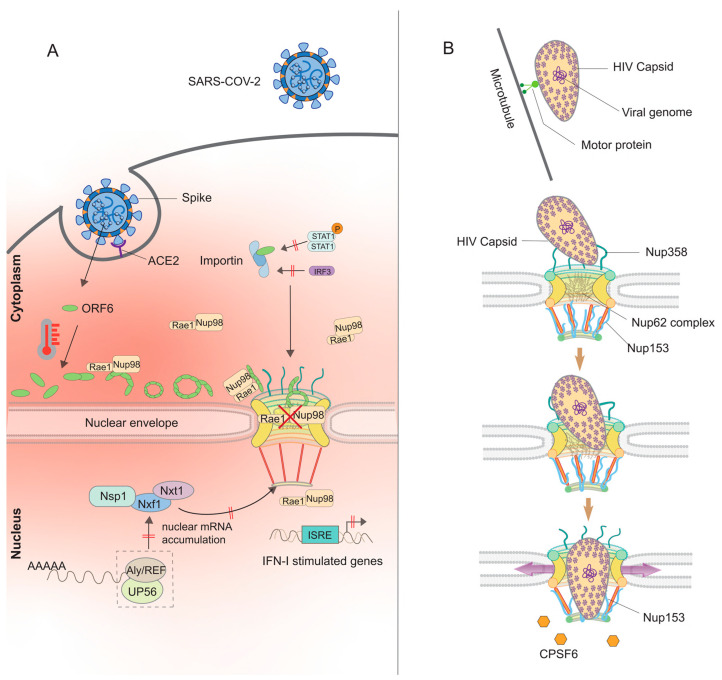
Interactions between viral components and the host nuclear transport system during infection. This schematic emphasizes the dual strategy of viral exploitation and host immune modulation within the nuclear transport system. (**A**) The accessory protein open reading frame 6 (ORF6) of severe acute respiratory syndrome coronavirus 2 (SARS-CoV-2) plays a critical role in suppressing host type-I interferon (IFN-I) signaling and contains amyloidogenic sequences. These sequences enable ORF6 amyloidogenic peptides to self-assemble into cytotoxic amyloid fibrils. ORF6 oligomers exhibit an ellipsoidal shape and readily assemble into protofilaments, forming either circular or linear patterns. This self-assembly process is enhanced at elevated temperatures or on lipid substrates. ORF6 self-assembly appears to play a dual role, both sequestering host factors and causing collateral cellular damage through the formation of amyloid aggregates. Previous studies, including ours, have demonstrated that Nup98 and Rae1 colocalize within ORF6 aggregates in cells. The combination of high body temperature and the localization of ORF6 oligomers on various membranous organelles significantly amplifies ORF6 intracellular aggregation. This aggregation effectively sequesters numerous host proteins, particularly transcription factors involved in IFN-I signaling, such as STAT1 and IRF3, thereby preventing their nuclear translocation and further suppressing immune responses. (**B**) The complete cone-shaped HIV-1 capsid, housing the viral RNA, travels along microtubules (MT) and motor protein through the cytosol toward the nuclear periphery. Upon reaching the NPCs, it engages with NUP358 on the cytoplasmic side and docks at the NPC via its slender, CA pentamer-rich end. The capsid then infiltrates the spiderweb-like central channel of the NPC. Structural modeling superimposing the HIV-1 capsid onto the in-cell NPC framework confirms that the central channel’s diameter is sufficiently wide to accommodate the full capsid, enabling its migration into the nucleus. HIV-1 capsid assemblies efficiently target NPCs in an NTR-independent manner, directly binding various FG-repeat types, including barrier-forming cohesive repeats. The capsid behaves analogously to an NTR, with its interior acting as a cargo container. This ‘self-translocating’ mechanism avoids the need for trans-acting NTRs, which would otherwise increase the capsid’s diameter by at least 10 nm, thereby circumventing the size restrictions imposed by the NPC scaffold and bypassing a critical barrier to infection.

Nishide et al. recently explored the structural dynamics of full-length ORF6 protein under near-physiological conditions using HS-AFM [97]. These findings reveal that ORF6 oligomers adopt an ellipsoidal shape and readily self-assemble into protofilaments, forming circular or linear patterns. The formation of these protofilaments was enhanced at elevated temperatures and on lipid substrates, suggesting a strong dependence on environmental factors. Biochemical analyses revealed that ORF6 filaments are sensitive to aliphatic alcohols, urea, and SDS, indicating that hydrophobic interactions predominantly stabilize their structure. This self-assembly mechanism likely underlies ORF6’s ability to sequester host factors, causing collateral cellular damage through the formation of amyloid aggregates (Figure 3).

These findings are significant in the context of neurological complications observed in some COVID-19 patients, which resemble Alzheimer’s disease (AD). Several SARS-CoV-2 proteins, such as spike and nucleocapsid proteins, have been implicated in disrupting the homeostasis of AD-associated proteins (e.g., tau, Aβ42, and α-synuclein), while other viral proteins, like ORF8, form aggregates in lung epithelial cells. Additionally, severe COVID-19 is often associated with excessive inflammation and elevated levels of amyloidogenic proteins, such as calprotectin (S100A8/S100A9). Together, these observations prompted us to investigate the amyloidogenic potential of ORF6 [97]. In silico analyses predicted a high propensity for spontaneous self-assembly of ORF6, a prediction supported by our HS-AFM observations of ORF6 protofilaments. The α-helix at the N-terminus of ORF6 appears to facilitate its localization on lipid membranes, where the fluidity of the lipid bilayer provides an ideal platform for ORF6 diffusion and robust protofilament elongation. Elevated body temperatures, a common symptom in COVID-19 patients, may further accelerate the formation and elongation of ORF6 protofilaments, supporting our model (Figure 3).

In cellular models, Kato et al. observed that ORF6 aggregates colocalize with Nup98 and Rae1 [93], supporting its role in sequestering host factors. ORF6 oligomers localize to membranous organelles, amplifying intracellular aggregation and effectively sequestering host proteins, particularly key transcription factors involved in type-I interferon signaling, such as STAT1 and IRF3. This sequestration impairs the nuclear translocation of these factors, undermining the host immune response. The Rae1/Nup98 complex may strategically locate Orf6 even inside the nuclear pore complex, modifying FG-Nup interactions and their capacity to facilitate nuclear transport [98]. Future studies, including cryo-EM imaging, could provide higher-resolution structural insights into ORF6 protofilaments, further elucidating the molecular basis of its aggregation and pathogenicity. These findings underscore the potential contribution of ORF6 to COVID-19 pathophysiology, particularly its role in immune evasion and cellular damage [97].

Orf6 also obstructs nuclear export by causing nuclear accumulation of hnRNPA1, a critical factor in mRNA export [93,99,100]. This suggests that Orf6 disrupts the Nup98–Rae1 interaction, impairing mRNA export. SARS-CoV-2 Nsp1 similarly inhibits mRNA export by interfering with the Nxf1–Nxt1 pathway and blocking interactions with adaptors like Aly/REF and UAP56 [101]. The acidic N-terminal patch in the Nsp1 was recently discovered to be critical for the interaction between Nsp1 and NXF1-NXT1 [102]. Unlike SARS-CoV-2 Nsp1, SARS-CoV-1 Nsp1 affects nucleoporin localization, selectively accumulating nuclear RNA-binding protein nucleolin in the cytoplasm, potentially hindering mRNA export [103]. SARS-CoV-2 Nsp14 uses its guanine-N7-methyltransferase active site to hijack the host association of the nuclear cap-binding complex (NCBC), thereby disrupting mRNA processing and export [104].

Other coronavirus proteins, such as SARS-CoV-1 Orf9b and MERS-CoV Orf4b, also interact with the host nuclear transport system. SARS-CoV-1 Orf9b lacks a NLS but passively enters the nucleus to activate caspase-3-mediated apoptosis. Its nuclear export, mediated by Crm1 through a NES, protects the host and enhances viral replication [105]. MERS-CoV Orf4b contains an NLS and interacts with importin-α3, inhibiting nuclear translocation of NF-κB p65 and suppressing IFN-β production by blocking IRF3 and IRF7 signaling [106,107].

#### 3.1.2. MonkeyPox (Mpox)

The monkeypox virus (MPXV) is an enclosed double-stranded DNA virus classified under the Orthopoxvirus genus in the Poxviridae family. MPXV induces the zoonotic affliction known as monkeypox (mpox) [108]. While both smallpox and mpox present comparable clinical symptoms, mpox has a reduced death rate compared to smallpox [108]. Recently, the incidence of mpox cases surged significantly, prompting the World Health Organization (WHO) to designate it a public health emergency of international concern [109]. MPXV exists in two distinct infectious forms: intracellular MPXV and extracellular MPXV, each expressing a diverse array of surface glycoproteins to infect host cells through varying methods [110]. In contrast to other DNA viruses, MPXV DNA replication transpires within viral factories situated near the perinuclear areas of the host cell [111]. MPXV encodes many viral elements to exploit the host’s nuclear transport mechanisms. The MPKV A23 protein, linked to apoptosis and DNA repair, possesses a nuclear localization signal (RKKR) that facilitates its localization within the host cell nucleus, where it interacts with ribosomal proteins and histones [112]. Another MPXV protein, P2, functions as an interferon antagonist and is also located in the nucleus [113]. The NLS within the P2 protein engages with karyopherin α-2 (KPNA2) for nuclear importation. The P2-KPNA2 interaction competitively obstructs KPNA2-mediated nuclear import of IRF3, thus diminishing interferon synthesis. Inhibition of the P2 protein reinstates host immunity and diminishes MPXV replication. Alongside MPXV proteins, host proteins contribute to the response to MPXV infection [114]. A recent study revealed that host proteins alter the NPC structure to mitigate MPXV infection. FAM111A (family with sequence similarity 111 member A), a host protein that regulates DNA replication, inhibits Orthopoxvirus infections. FAM111A degrades NPC constituents, including Nup62, to translocate to the cytoplasm, where it degrades the viral DNA-binding protein I3 via autophagy. Conversely, SPI-1 proteins produced by Orthopoxviruses exhibit a significant affinity for FAM111A, inhibiting its function and promoting viral multiplication within host cells [114].

#### 3.1.3. Chikungunya Virus (CHIKV)

The Chikungunya virus (CHIKV), belonging to the Togaviridae family, is an enveloped virus characterized by a positive-sense, single-stranded RNA genome. It exclusively replicates in the cytoplasm of host cells, employing the host’s translational machinery to synthesize viral proteins and form progeny virions. CHIKV is predominantly transmitted to humans via Aedes mosquitoes, specifically Aedes aegypti and Aedes albopictus, and has resulted in substantial outbreaks in tropical and subtropical areas. CHIKV infection is primarily linked to fever sickness, rash, and severe arthralgia during the acute phase. A defining characteristic of CHIKV infection is its tendency to induce chronic inflammatory arthritis and enduring musculoskeletal problems, which may last for months or even years in certain patients. The chronic symptoms, often known as Chikungunya arthritis, are thought to arise from immune-mediated processes, such as the retention of viral RNA in joint tissues and dysregulated inflammatory responses. Comprehending CHIKV’s pathophysiology and its interactions with the human immune system is essential for devising effective therapies and prevention strategies [115]. The CHIKV capsid protein (CP) possesses two NESs and one NLS [116,117]. CP specifically associates with the C-terminal NLS-binding domain of importin-α3 for nuclear translocation [116]. It engages with Crm1 via a nuclear export signal situated within a leucine-rich region (amino acid residues 143–155), directing importin-α3 and Crm1 to influence host nucleocytoplasmic transport. The mutation of the CP NES at the N-terminus (residues 44–53) results in the nuclear retention of CP and obstructs the host’s nuclear import mechanism for undetermined reasons [117].

CHIKV nonstructural protein 2 (nsP2) is instrumental in mitigating the host’s IFN-induced antiviral response [118,119,120,121]. Although nsP2 does not possess a typical NLS, its nuclear import is crucial for its interferon-antagonistic actions [121]. Analogous to DENV NS3, nsP2 undergoes transitory nuclear localization during the initial stages of infection, thereafter relocating to the cytoplasm as the infection advances. nsP2 employs multiple mechanisms to suppress IFN signaling and establish a favorable environment for viral replication. These include translational repression, which reduces levels of cyclic GMP-AMP synthase (cGAS), a key sensor in the host’s innate immune system [118,122]. Additionally, nsP2 diminishes the activation of STAT1 and impairs the nuclear import of pY-STAT1, a crucial transcription factor for IFN-stimulated gene expression [120]. Furthermore, nsP2 promotes the nuclear export of STAT1, effectively disrupting its ability to sustain an antiviral response [119]. These strategies underscore nsP2’s central role in CHIKV’s immune evasion and highlight its potential as a target for antiviral therapies.

#### 3.1.4. Dengue Virus (DENV) and Zika Virus (ZIKV)

Flaviviruses, belonging to the Flaviviridae family, are diminutive, enveloped, positive-sense RNA viruses that reproduce within vesicular packets on the endoplasmic reticulum. The dengue virus (DENV), a prominent mosquito-borne flavivirus in tropical and subtropical areas, induces dengue illness. The DENV nonstructural proteins NS3 helicase and NS5 RNA-dependent RNA polymerase (RdRp) constitute a complex crucial for viral replication [123,124]. NS5 obstructs IFN-induced antiviral responses by promoting the degradation of STAT2 [125]. It has a bipartite NLS, including a βNLS for binding to importin-β1 and a cNLS for binding to importin-α/β1 [126,127]. Nuclear import is chiefly facilitated by importin-α/β1; nevertheless, subsequent studies revealed that these NLSs are inaccessible to host importins [128]. The subcellular localization of NS5 differs across DENV serotypes: NS5 from DENV2 and DENV3 is located in the nucleus, whereas NS5 from DENV1 and DENV4 is found in the cytoplasm [129,130]. A recently discovered monopartite nuclear localization signal at the C-terminus of NS5 is associated with robust importin-α2 binding in DENV2/3, while showing diminished interactions in DENV1/4 [130]. The nuclear export of NS5 through Crm1 is associated with elevated IL-8 production and reduced viral replication, but the underlying mechanism remains ambiguous [131]. Abnormalities in the nuclear envelope and reduced integrity of the NPC have been noted in DENV-infected cells, with NS3 and its cofactor NS2B3 facilitating the proteolytic degradation of FG-nucleoporins, such as Nup62, Nup153, and Nup98 [132]. NS3 possesses putative NLS and NES, initially localizing to the nucleus during early infection stages and then migrating to the cytoplasm [133].

The Zika virus (ZIKV), a member of the Flaviviridae family, has attracted attention due to its correlation with microcephaly in infants born to infected women [134,135]. Like DENV, ZIKV utilizes host NTRs and Nups via its NS3 helicase and NS5 RdRp. ZIKV NS3, along with NS2B3, destroys FG-rich nucleoporins, including TPR, Nup153, and Nup98, hence compromising NPC integrity and the structure of the nuclear envelope [132]. ZIKV NS5 possesses a bipartite nuclear localization signal (βNLS and cNLS) that is acknowledged by importin-α7, enabling its nuclear translocation and safeguarding against cytoplasmic degradation, hence enhancing viral replication [136,137]. Although the cNLS was first regarded as the principal NTR binding site [138], both βNLS and cNLS are essential for nuclear import [87]. Upon entering the nucleus, NS5 sequesters α-importins (α1, α3, and α4) within nuclear bodies, interacting with STAT1 in glioblastoma cells, but not in hepatocellular carcinoma cells, indicating tissue-specific functions in inflammatory responses [138]. In contrast to the NS5 protein of the Japanese encephalitis virus (JEV), which competes with host IRF3 and NF-κB for α-importins [139], ZIKV NS5 inhibits TANK-binding kinase 1, hence obstructing IRF3 activation and interferon generation [140]. ZIKV NS2A further promotes chaperone-mediated autophagy of importin-α1, perhaps inhibiting host antiviral responses.

#### 3.1.5. Ebola Virus (Zaire Ebolavirus)

The Ebola virus (EBOV), belonging to the Filoviridae family, is a filamentous, enveloped, single-stranded, negative-sense RNA virus. Six species of EBOV have been found, with the Zaire ebolavirus species responsible for two outbreaks in Africa, leading to fatality rates of 40% and 66%. EBOV replicates in the cytoplasm and utilizes the host’s nuclear transport system to circumvent IFN-mediated antiviral responses.

The viral protein VP24 selectively interacts with importin-α5, -α6, and -α7, but not with importin-α1, -α3, or -α4 [141,142]. VP24 identifies a non-classical NLS-binding site in importin-α6, allowing EBOV to inhibit the nuclear translocation of pY-STAT1 and reduce interferon signaling, including the generation of IFN-λ [142,143,144]. VP24 additionally associates with unphosphorylated STAT1 (U-STAT1), which translocates to the nucleus autonomously, bypassing importins, through Nup153 and Nup214 to modulate particular IFN-induced genes [145,146,147]. The interaction of VP24 with importin-α5 results in the cytoplasmic accumulation of hnRNP C1/C2, a nuclear protein implicated in IRES-dependent c-myc translation during mitosis [148,149]. Comparable translocation of hnRNP C1/C2 to the cytoplasm has been documented in other viruses to facilitate replication or IRES-mediated protein synthesis [150,151,152,153], underscoring its essential function in EBOV replication.

EBOV replication transpires within inclusion bodies (IBs), constituted by nucleoproteins (NPs) and VP35 [154]. Importin-α7 is crucial for IB development, promoting genomic RNA replication [155]. Alongside genomic RNA, sub-genomic EBOV mRNAs are generated for the synthesis of viral proteins. EBOV NP engages with Nxf1, altering the RNA-binding affinity of Nxf1 from nucleoporins to mRNA. This association facilitates the export of viral mRNA to the cytoplasm for translation [156].

### 3.2. Viruses That Replicate in the Nucleus

#### 3.2.1. Human Immunodeficiency Virus (HIV)

The human immunodeficiency virus (HIV), belonging to the Retroviridae family, is an encapsulated, positive-sense, single-stranded RNA virus that was initially identified in 1981 as the causative agent of a new disease affecting T cells. HIV-1, which is more virulent and infectious than HIV-2, is the primary etiological agent of acquired immunodeficiency syndrome (AIDS) in the current epidemic [157]. After entering the host cell, the intact, cone-shaped HIV-1 capsid, containing the viral DNA, traverses microtubules and motor proteins across the cytoplasm towards the nuclear perimeter [158,159,160,161]. The viral replication complex performs reverse transcription, resulting in the formation of a pre-integration complex (PIC) that facilitates nuclear entry [162]. Mutations in HIV proteins and the silencing of certain Nups affect the selection of integration sites for viral cDNA, highlighting the significance of nuclear import in viral replication and host genome targeting [163].

Initially, it was thought that the HIV-1 capsid disassembled quickly upon entry, facilitating the release of the PIC for nuclear import via the NPC [164]. Recent investigations indicate that the HIV-1 capsid remains intact during reverse transcription and may only uncoat upon entering the nucleus [165,166,167,168]. Upon arriving at the NPCs, it interacts with NUP358 on the cytoplasmic side and attaches to the NPC through its elongated, CA pentamer-rich terminus. The capsid subsequently infiltrates the spiderweb-like core channel of the NPC. Advanced imaging methodologies, including cryo-electron tomography (cryo-ET) and subtomogram averaging, have demonstrated that the complete HIV-1 capsid may traverse the NPC in its entirety [169]. This discovery contradicts the previous idea that capsid breakdown was essential for nuclear import. The diameter of the nuclear pore in a transport-ready condition surpasses the wider end of the capsid (~60 nm), facilitating complete capsid entry. Zila and colleagues suggested a three-phase model for capsid nuclear import, in which the intact capsid successively interacts with several Nups. During the initial phase, the capsid interacts with FG-repeats and the cyclophilin (Cyp) domain of Nup358 on the cytoplasmic aspect of the NPC. Xue et al. additionally found that both Nup35 and POM121 assist in the nuclear entrance of the HIV capsid following its interaction with Cyp [170].

During the second phase, it navigates the NPC core, engaging with FG-rich Nup62 complexes. In the last phase, the capsid interacts with Nup153 and cleavage and polyadenylation specificity factor subunit 6 (CPSF6), initiating uncoating and releasing the PIC into the nucleoplasm. Structural study of the docked entire capsids revealed connected striated patterns of lattice disorder, possibly associated with the intrinsic elasticity of the capsid. The presence of uncondensed genomic material within the docked capsid increases the overall lattice disorder of the capsid. This indicates that innate “elasticity” can assist the capsid in adapting to stress while maintaining structural integrity during translocation.

Furthermore, recent findings indicate that the HIV capsid functions as a nuclear transport receptor, facilitating its passage through the nuclear pore complex by engaging with the FG domains of nucleoporins [171,172,173]. The capsid functions similarly to a nuclear transport receptor, with its interior serving as a cargo container. This ’self-translocating’ method eliminates the necessity for trans-acting NTRs, which would otherwise augment the capsid’s width by a minimum of 10 nm, thus evading the size constraints imposed by the NPC scaffold and overcoming a significant obstacle to infection (Figure 3).

HIV also alters host nuclear transport mechanisms to escape immune responses and promote replication. Viral protein R (Vpr) impedes host antiviral mechanisms by binding to α-importins (mostly α5 and, to a lesser degree, α1 and α4), thus obstructing IRF3 activation and its nuclear import, along with the nuclear translocation of NF-κB [174]. Vpr includes NLSs at both the N-terminal and C-terminal regions.

HIV-1 Rev is essential for the export of unspliced viral RNA (vRNA) to the cytoplasm for translation [175]. Rev contains an arginine-rich NLS in its N-terminal domain, facilitating importin-β1-mediated nuclear import, and a leucine-rich NES in its C-terminal area. Rev binds vRNA via the Rev response element (RRE) and engages with Crm1 in a RanGTP-dependent fashion to facilitate the export of vRNA as a viral ribonucleoprotein (vRNP) complex. The export mediated by Rev entails indirect contacts between vRNP and nucleoporins, including Nup214, Nup153, Nup98, and Nup62, facilitated by Rev cofactors [5,176,177,178].

Additionally, Rev obstructs Nxf1-mediated RNA export, hence facilitating appropriate viral gene expression by preventing premature translation of viral transcripts [175]. This suppression facilitates HIV persistence despite antiretroviral therapy, as Crm1 and Rev-mediated export of unspliced HIV transcripts can induce persistent inflammation in infected individuals [179].

These findings collectively highlight the complex pathways via which HIV-1 exploits host nucleocytoplasmic trafficking to facilitate its reproduction, evade the immune response, and maintain survival, while concurrently altering host nuclear transport to promote its lifecycle. Additional investigation into these mechanisms may yield novel pharmaceutical targets for the effective treatment of HIV infection [5,176,177,178].

#### 3.2.2. Influenza A Virus (IAV)

Influenza A virus (IAV), belonging to the Orthomyxoviridae family, is an enveloped, segmented, negative-sense RNA virus responsible for pandemic respiratory diseases. Intranuclear routes are used for viral gene expression as IAV proliferate within the host cell nucleus. Two of the eight genomic vRNP segments of IAV generate viral mRNAs (M and NS) that undergo alternative splicing before being exported from the nucleus [180]. IAV genomic RNAs are encapsulated with a viral nucleoprotein (NP) and a heterotrimeric RNA-dependent RdRp complex, consisting of PA, PB1, and PB2, into rod-shaped vRNPs [181]. After entry, free vRNPs are transported to the nucleus for transcription and replication of viral RNA. Both the NP and the RdRp components possess NLSs essential for nuclear import [182]. IAV NP contains two NLSs: a non-classical N-terminal NLS [183] and a centrally located bipartite classical NLS (cNLS) [184], both sufficient for vRNP nuclear import. Mutations in the bipartite cNLS do not prevent nuclear import, indicating that the non-classical NLS, which binds importins α5 and α7, is the primary driver of nuclear transport and critical for replication [185].

PB2, a component of the RdRp, possesses a conventional bipartite NLS that interacts with α-importins (α1, α5, and α7), with a preference for importin-α7 [186,187,188,189]. Importins α1 and α7 enhance PB2 polymerase activity, while importin-α3 acts as a negative regulator [188]. Highly pathogenic avian influenza viruses (HPAIVs) with human-like PB2 reduce importin-α3 expression, inhibiting the nuclear transport of NF-κB and weakening host antiviral responses [190]. Furthermore, the importin-β family member IPO5 (RanBP5) establishes an import complex with PA and PB1, modulating PA–PB1 interactions for vRNA binding [191,192].

The Crm1-facilitated nuclear export of newly synthesized vRNPs is an essential phase in the replication cycle of numerous viruses. The export process guarantees the efficient transfer of vRNPs, composed of viral RNA encapsulated by nucleoproteins, from the nucleus to the cytoplasm, where future phases of the viral lifecycle, such as assembly and budding, take place. The nuclear export of vRNPs is enabled by a multiprotein complex that includes vRNP, matrix protein 1 (M1), and nonstructural protein 2 (NS2) [193,194]. The host Ran protein enhances the binding affinity of Crm1 to the IAV NS2 protein, thereby promoting the nuclear export of vRNP [195]. IAV NS1 suppresses host mRNA export by diminishing Nup98 expression and creating an inhibitory complex with mRNA export factors including Rae1, Nxt1, Nxf1, and E1B-AP5 [196]. In severe infections, reduced PD1 levels enable direct binding of IAV vRNA to Nxf1, facilitating nuclear export through interactions with Nup62, bypassing the need for mRNA export factors (e.g., Nxt1, Crm1) or FG-containing nucleoporins (e.g., Nup98, Nup214) [197]. In addition to IAV proteins, the virus also exploits the host factor, Makorin-2 (MKRN2), to facilitate IAV mRNA export potentially through its association with the RNA export mediator GLE1 [198].

#### 3.2.3. Human Papillomavirus (HPV)

The human papillomavirus (HPV), a member of the Papillomaviridae family, is a small, icosahedral, non-enveloped virus with a double-stranded DNA genome. HPV has garnered significant attention due to its strong association with various cancers, particularly cervical cancer. In 1995, the International Agency for Research on Cancer (IARC) classified HPV16 and HPV18 as human carcinogens, recognizing their critical role in the etiology of cervical cancer and other anogenital [199] and oropharyngeal malignancies [200]. Unlike many DNA viruses that utilize NPCs for nuclear entry, HPV takes a distinct approach to accessing the host nucleus. During mitosis, as the nuclear envelope disintegrates, the viral genome takes advantage of the transient loss of the nuclear barrier to gain entry into the nucleus. Once inside, HPV replicates its genome and hijacks the host’s transcriptional machinery to express viral proteins, which contribute to viral replication and oncogenesis. This unique nuclear entry mechanism highlights HPV’s adaptation to the cellular environment and underscores its reliance on host cell division for successful infection and replication. These characteristics make HPV a compelling target for preventive measures, including vaccines, and therapeutic interventions aimed at mitigating its oncogenic potential [201,202].

The nucleocytoplasmic transport of the oncoproteins E6 and E7 in high-risk HPVs drives carcinogenesis. High-risk E6 (E6high) localizes to the nucleus due to three NLSs in its C-terminus, interacting with importin-α1/β1, importin-β1, and importin-β2, while low-risk E6 (E6low) remains primarily in the cytoplasm unless fused with an E6high NLS [203]. Nuclear E6high facilitates p53 degradation via polyubiquitination, independent of MDM2, but requires the p53 NES for Crm1-mediated nuclear export [204].

High-risk E7 (E7high) translocates to the nucleus to reprogram the host cell cycle, inducing S-phase re-entry for viral DNA amplification [198]. While initially thought to lack an NLS, HPV16 E7 contains two NLSs in its N-terminal and CR3 domains [205]. Nuclear import occurs independently of importins but involves hydrophobic interactions with FG-domains of Nup62 and Nup153 [206,207]. This hydrophobic interaction is conserved across other HPV types, including HPV8 and HPV11 [207,208]. A functional NES allows Crm1-dependent nuclear export of E7 [205].

HPV encodes E1 DNA helicase and E2 origin recognition proteins to hijack host DNA replication machinery. E1 proteins possess bipartite NLSs and interact with importins α1, α3, and α5 for nuclear import, activated by host ERK or JNK kinases [209,210]. E1 also contains a Crm1-dependent NES, regulated by CDK phosphorylation, ensuring its nuclear retention for viral replication [210,211]. E2 localization varies between low- and high-risk HPVs, with low-risk E2 primarily nuclear, while high-risk E2 shuttles between the cytoplasm and nucleus [212]. High-risk E2 NES-mediated cytoplasmic accumulation induces caspase-8-mediated apoptosis, highlighting its role in regulating cell death [212].

Capsid proteins L1 and L2 play essential roles in the assembly of HPV virions, mediating key processes for viral genome encapsidation and infectivity. HPV16 L1, the major capsid protein, contains both monopartite and bipartite NLSs that facilitate its nuclear import. This process occurs through an importin-α1/β1-dependent pathway, requiring the RanGDP/GTP cycle but notably independent of GTP hydrolysis [213]. Additionally, L1 can bind directly to importin-β2; however, this interaction is distinct in that RanGTP fails to dissociate the L1-importin-β2 complex, a mechanism that can interfere with the nuclear import of other cargoes, such as hnRNP A1 [213]. The nuclear import of L2, the minor capsid protein, is equally critical for successful virion assembly. L2 interacts with several nuclear transport receptors, including importin-β2, importin-5, and importin-α1/β1 heterodimers, to gain access to the nucleus. Interestingly, L2 also harbors a NES, but the functional role of this NES remains unclear [214]. These intricate interactions between capsid proteins and the host nuclear transport machinery underscore the sophisticated strategies HPV employs to hijack cellular pathways for efficient virion assembly and highlight potential therapeutic targets for disrupting HPV replication and assembly.

#### 3.2.4. Hepatitis B Virus (HBV)

The Hepatitis B virus (HBV), belonging to the Hepadnaviridae family, is a diminutive, encased, double-stranded DNA virus that replicates in hepatocytes. Acute HBV infection can result in acute hepatitis, characterized by liver inflammation, jaundice, and elevated liver enzyme levels. While many individuals clear the virus and recover fully, in a subset of cases, HBV infection persists, transitioning into a chronic state. Chronic HBV infection significantly increases the risk of developing hepatocellular carcinoma (HCC), the most common form of primary liver cancer. This elevated risk arises from the cumulative effects of ongoing liver inflammation, viral integration into the host genome, and the disruption of normal cellular processes, which collectively promote genomic instability and tumorigenesis. Understanding the mechanisms underlying HBV persistence and its link to HCC is crucial for improving prevention, diagnosis, and treatment strategies [215]. Within the nucleus, the HBV capsid disassembles, releasing relaxed circular DNA (rcDNA). The host’s DNA repair machinery converts rcDNA into covalently closed circular DNA (cccDNA), which serves as the template for viral RNA transcription [216].

Phosphorylation of the C-terminal HBV core/capsid protein (Cp) exposes its NLS, allowing recruitment of classical importin-α/β1 receptors to mediate nuclear import [217,218]. Additionally, the synthesis of (+) DNA and removal of viral DNA polymerase from rcDNA induce a conformational change in Cp, revealing its C-terminal NLS [219]. During nuclear translocation through the NPC, RanGTP dissociates the Cp-importin-α/β1 complex, enabling Cp interaction with Nup153. This interaction, distinct from hydrophobic mechanisms, is specific to Nup153, the only FG-containing nucleoporin engaged by Cp [220]. Mature HBV capsids disassemble in a Ran-independent manner to release rcDNA, while immature capsids fail to disassemble.

Cp contains four arginine-rich domains (ARDs): ARD I and III function as NLS-like regions, while ARD II and IV act as NES-like regions [221]. The Cp NES is critical for Nxf1-mediated export of pre-genomic RNA (pgRNA), an essential step for virion production [221]. Cp subcellular localization is regulated by intrinsic factors (NLS and NES) and extrinsic factors (importins, Nxf1, and cellular kinases) [221]. Elevated nuclear Cp concentrations promote Crm1-dependent export, suggesting that Cp concentration influences export signaling [222]. Recent studies have revealed that HBV exploits the host Crm1 to export its capsid containing pgRNA [223,224]. Two NES were found at the spike tips of HBV capsid particles [224]. However, HBV capsid is not essential for Crm1-mediated pgRNA nuclear export because another host factor, the Embryonic lethal, abnormal vision, Drosophila-like 1 (ELAVL1, or HuR) protein, binds directly to pgRNA [223]. ELAVL1 then recruits ANP32A and ANP32B, which contain leucine-rich NES, to facilitate the export of pgRNA to the cytoplasm via the Crm1-mediated pathway [223]. Additionally, unbound Cp can interact directly with importin-β1 via its IBB domain, though the biological relevance of this interaction remains unclear [225].

The hepatitis B virus e antigen (HBeAg), also referred to as the precore protein intermediate (p22), plays a pivotal role in modulating the host immune response. Through its C-terminal arginine-rich domain (ARD), HBeAg interacts specifically with importin-α5, a key nuclear transport receptor. This interaction inhibits the nuclear import of pY-STAT1, a critical transcription factor involved in IFN-mediated antiviral signaling. By blocking pY-STAT1 translocation to the nucleus, HBeAg effectively suppresses the activation of interferon-stimulated genes (ISGs), thereby dampening the host’s antiviral defense mechanisms. This immune evasion strategy highlights the sophisticated ways in which HBV undermines host immunity to establish persistent infection and underscores the importance of HBeAg in HBV pathogenesis [226]. This highlights the multifaceted strategies HBV employs to manipulate host nucleocytoplasmic trafficking for immune evasion and replication.

#### 3.2.5. Herpes Simplex Virus Type-1, Human Cytomegalovirus, and Epstein–Barr Virus

Herpes Simplex Virus Type 1 (HSV-1):

HSV-1, a double-stranded DNA α-herpesvirus, causes cold sores in humans [227]. Subsequent to internalization, the viral capsid uses microtubules to reach the nucleus, where it releases viral DNA for transcription and replication [228]. The tegument proteins VP1/2 and pUL25 play critical roles in herpesvirus infection by mediating the docking of the viral capsid onto the NPC. These proteins interact with key NPC components, such as Nup358 and Nup214, to ensure precise capsid positioning at the nuclear envelope, a prerequisite for successful viral genome delivery into the nucleus. VP1/2 contains an N-terminal NLS that facilitates its interaction with importin-β1, a major nuclear transport receptor. Interestingly, this NLS does not engage with importin-α, highlighting a unique mechanism of NPC targeting. This importin-β1-mediated docking mechanism enables the viral capsid to exploit the host’s nucleocytoplasmic transport machinery for efficient nuclear entry, underscoring the critical role of VP1/2 and pUL25 in the herpesvirus lifecycle [229,230]. While importins are not required for capsid docking or genome import, they modulate viral protein nuclear localization and production [231]. The DNA polymerase subunits pUL30 and pUL42 use multiple nuclear import pathways, with pUL30 interacting with importin-α5 and pUL42 primarily binding importin-α7 [232,233,234,235]. Capsid assembly and egress rely on importin-α1, as silencing importin-α1 or α3 inhibits HSV-1 gene expression in differentiated cells like neurons [231].

HSV-1 ICP27, an immediate-early protein, enhances viral gene expression and mediates the export of intronless HSV-1 mRNAs by recruiting Aly/REF and Nxf1 [236,237,238]. Reduction of Aly/REF does not inhibit mRNA export, suggesting alternative pathways [239]. ICP27 interacts with Nup62 for nucleocytoplasmic shuttling, bypassing traditional importin- or transportin-dependent pathways [240,241].

Human Cytomegalovirus (HCMV):

HCMV, a β-herpesvirus, causes severe complications in immunocompromised individuals, including mononucleosis, thromboembolism, and encephalitis. Hong and colleagues reported that HCMV and HSV-1 hijacked host immune adaptor stimulator of interferon genes (STING) for nuclear import of viral genome [242]. Later, Lee and coworkers found that host YES-associated protein (YAP) negatively regulates HCMV replication by downregulating STING expression [243]. HCMV pUL44, a viral DNA polymerase processivity factor, expresses a classical NLS to exploit host importin-α for nuclear translocation, which is inhibited by phosphorylation [244]. HCMV pUL97, a kinase with two isoforms, manipulates host cell-cycle regulators for viral replication. The larger isoform has two bipartite NLSs, enabling superior nuclear localization compared to the smaller isoform, which has only one NLS [245,246]. pUL79, an elongation factor for RNA polymerase II, uses a PY-NLS for importin-β2-mediated nuclear import [247,248]. HCMV pUL84, essential for lytic DNA synthesis, depends on importin-α isoforms (α1, α3, α4, and α5) for nuclear import and has two Crm1-dependent NESs for export [249,250,251]. pUL69, akin to HSV-1 ICP27, facilitates the export of intronless viral mRNAs by interacting with UAP56/URH49 and Nxf1-Nxt1. CDK9 phosphorylation of pUL69 is critical for its mRNA export function [252,253].

Epstein–Barr Virus (EBV):

EBV, a γ-herpesvirus, is linked to lymphomas (e.g., Burkitt’s, Hodgkin’s) and nasopharyngeal carcinoma [254]. During latency, EBNA1 facilitates viral DNA retention in the nucleus via its NLS, which interacts with importin-α1 and α5 [255]. During lytic replication, viral proteins (e.g., BSLF1, BBLF2/3, BBLF4, VCA) lacking NLSs require BGLF4 to promote nuclear import by phosphorylating FG-Nups (Nup62, Nup153) and reorganizing microtubules [256]. BGLF4 homologs in other herpesviruses, such as HSV-1 UL13 and HCMV UL97, also facilitate nuclear lamina disassembly [257,258].

EBV mRNA export relies on EB2 (SM protein), which interacts with Crm1, Ran, and Nup214 to transport unspliced viral mRNA [259]. Nuclear egress proteins BFLF1 and BFRF2 form a nuclear egress complex (NEC) in the inner nuclear envelope to export viral capsids [260,261]. BFLF2 interacts with Nxf1 for RNA-independent export but does not require Crm1. Functional NESs in BFLF2 resemble those in HSV-1 UL31, which also uses Ran, importin-α1, and importin-β2 for nuclear import [262].

Tegument Protein Export:

HSV-1 tegument proteins possess diverse export mechanisms. While HSV-1 pUL21 and pUL48 rely on Crm1-dependent export, others like pUL14 are Crm1-independent. EBV tegument proteins pBTRF1 and pBGFL3 use Rev-like and PKI-like NESs, respectively, for Crm1-dependent export [263]. These varied export mechanisms reflect the adaptability of herpesviruses in nucleocytoplasmic transport.

## 4. Targeting Host Nuclear Transport Machinery: Potential Antiviral Strategies

The exploitation of host nucleocytoplasmic trafficking pathways is essential for viral replication, enabling genome replication, assembly of viral components, suppression of antiviral immune responses, and modulation of the host cellular environment to favor infection. In clinical settings, viral polymerase inhibitors are widely used to combat RNA and DNA viruses, including Ebola virus (EBOV) [264], human immunodeficiency virus (HIV) [265], and human papillomavirus (HPV) [266]. While these inhibitors have shown considerable efficacy, targeting nuclear transport pathways offers an alternative therapeutic approach.

Nuclear transport inhibitors aim to disrupt the trafficking of viral components or modulate host nuclear transport machinery to reduce viral replication and improve clinical outcomes. These inhibitors can be employed as standalone therapies or in combination with other antiviral agents, such as polymerase inhibitors, to achieve synergistic effects. This section delves into both host-specific and virus-specific nuclear transport inhibitors, highlighting their mechanisms of action, potential benefits, and ongoing clinical trials evaluating their effectiveness in treating viral infections. These strategies represent a promising avenue for expanding the arsenal of antiviral therapies.

### 4.1. Targeting Host Nuclear Import Pathways: Specific Inhibitor Strategies

The importin-α/β1 heterodimer is a key nuclear transport receptor exploited by many viruses. Inhibitors targeting importin-α include Bimax (1 and 2) [267], cSN50.1 [268,269], Ivermectin [270,271], and GW5074 [272]. Of these, cSN50.1 selectively inhibits importin-α5 [269], while Ivermectin and GW5074 block cargo loading and heterodimer assembly by binding importin-α. Ivermectin exerts broad-spectrum antiviral activity, including against SARS-CoV-2 [273], while GW5074 shows efficacy against DENV2 and ZIKV in vitro [273].

Importin-β inhibitors prevent the formation of the cargo-receptor ternary complex. Notable inhibitors include Importazole [274], INI-43 [275], Karyostatin [276], and M9M (specific to importin-β2) [277]. Among these, only M9M has been evaluated for antiviral activity, successfully blocking HSV-1 UL6 nuclear import [278].

### 4.2. Host-Specific Nuclear Export Inhibitors

Crm1 is a crucial target for viral nuclear export. Leptomycin B (LMB) and its derivatives [279,280], along with selective inhibitors of nuclear export (SINE) [281,282], target Crm1’s Cysteine-528 site. LMB irreversibly inhibits Crm1 by forming a salt bridge [283], leading to adverse effects in clinical trials [284]. Conversely, SINEs, such as Selinexor and Verdinexor, inhibit Crm1 reversibly, reducing toxicity [285]. As Crm1 is crucial for the nuclear export of a vast number of cargo types, it is a main target.

### 4.3. Inhibitors of Viral-Specific Nuclear Transport

*N*-(4-hydroxyphenyl) retinamide (4-HRP) inhibits Flavivirus polymerase NS5 nuclear import, indicating efficacy against DENV and ZIKV while preventing antibody-dependent enhancement (ADE) in dengue hemorrhagic fever [286,287,288]. In neurons, 4-HRP blocks ZIKV NS5 nuclear accumulation, potentially reducing neurological complications [138].

SARS-CoV-2 Orf6 oligomerization could disrupt host nucleocytoplasmic trafficking. Nishide et al. demonstrated that LLPS inhibitors, such as 1,6-hexanediol (1,6-HD) and cyclohexanediol (CHD), promoted disassembly of Orf6 oligomers by disrupting hydrophobic interactions [97]. Inhibition of SARS-CoV-2 guanine-N7-methyltransferase by small molecules [289,290] not only suppresses viral replication but could also restore host mRNA exports.

HIV capsid inhibitors GS-6207 and GS-CA block viral PIC nuclear entry by targeting the capsid at Nup153 and CPSF6 interaction sites, disrupting capsid assembly and replication. The results of clinical trials indicated that GS-6207 provides long-lasting suppression of HIV viral load [291]. In addition to inhibiting the interaction of capsid with host factors, capsid inhibitors could also reduce its elasticity and prevent the nuclear entry of HIV capsid [292]. Targeting the NLS of HIV-1 integrase with GRL-42 to inhibit nuclear import of the HIV-1 pre-integration complex (PIC) is also a promising strategy to combat HIV-1 infectivity and replication [293].

For IAV, Mohl et al. identified inhibitors targeting PB1/PA-RanBP5 interactions, impairing polymerase activity and nuclear localization in vitro [294]. The inhibition of nuclear import and export of IAV ribonucleoproteins has been actively investigated to develop new therapeutics for halting IAV replications. Examples include CDK9 inhibitor (LDC000067) [295], viral nuclear export protein peptide mimics [296], sodium polyoxotungstate [297], NRF2 activators [298,299], chalcone-like derivatives [300,301], and Artesunate [302]. For EBOV, oligonucleotide-based aptamers (VPKS-2 and VPKS-5) and macrocyclic peptides inhibit VP24 interactions with importins α1 and α6, restoring host antiviral defenses [303,304].

In HPV, recombinant p56 protein inhibits E1 helicase activity, blocking viral DNA replication and promoting E1 nuclear export [305,306]. HBV capsid assembly modulators (CAMs), including HAPs, PPAs, and SBAs, prevent nuclear entry and disrupt cccDNA production, reducing chronic infection and viral load [307,308,309].

### 4.4. Advancing Nuclear Transport Inhibitors for Antiviral Therapeutics

Ivermectin has undergone clinical trials for antiviral efficacy. Results suggest that Ivermectin has no clinical value for early-diagnosed COVID-19 patients [310], mild to moderate COVID-19 cases [311], reducing of long COVID-19 incidences [312], or lowering of SARS-CoV-2 viral load [313]. Ivermectin has also shown potential in clearing NS1 antigenemia in dengue fever, though its role in preventing severe disease remains unexplored [314]. Various clinical trials highlight the reduced toxicity of SINE inhibitors Selinexor and Verdinexor when compared to LMB. Selinexor is under evaluation for treating COVID-19, while Verdinexor has shown promise in preclinical studies [281,284]. Both SINEs are under evaluation for antiviral efficacy, with Selinexor showing promise in moderate-to-severe COVID-19 cases.

Among viral-specific inhibitors, Lenacapavir (GS-6207) significantly reduced HIV viral load in clinical trials [291]. Moreover, two clinical studies reported that Lenacapavir is also effective in suppressing multidrug-resistant HIV-1 viruses [315,316]. REBACIN^®^, evaluated in HPV patients, inhibits E6 and E7 mRNA transcription and has shown superior efficacy compared to interferon in persistent high-risk HPV infections [317,318]. For HBV, CAMs such as NVR 3-778 and GLS4 have exerted antiviral activities, reducing viral DNA and RNA levels with minimal side effects [319,320].

## 5. Conclusions

Viral proteins often mimic host cellular factors to hijack nuclear transport mechanisms, a strategy that enables them to efficiently replicate within host cells. By targeting the nuclear transport machinery, these viral proteins manipulate nucleocytoplasmic trafficking to deliver viral genomes, enzymes, and other components to specific subcellular compartments. In addition to facilitating replication, this mimicry allows viruses to reprogram host cellular processes, including gene expression and signal transduction, to create an environment conducive to their lifecycle. Simultaneously, these strategies help viruses evade host immune defenses by disrupting key pathways, such as interferon signaling and the nuclear translocation of antiviral transcription factors. This dual functionality underscores the sophisticated mechanisms viruses use to exploit host systems for survival and propagation. In addition to affecting nuclear transport, certain viral genomes behave like oncogenes, functioning as enhancers [321] to facilitate viral replications [322] or induce oncogenesis [323]. Nuclear transport inhibitors have emerged as a promising class of therapeutics capable of targeting key processes in viral lifecycles. By disrupting the nucleocytoplasmic trafficking pathways exploited by viruses, these inhibitors can effectively halt viral replication, preventing the transport of viral genomes, proteins, or ribonucleoprotein complexes to their intended subcellular destinations. Additionally, nuclear transport inhibitors can impair the assembly of viral components by blocking their nuclear import or export, thereby interfering with virion formation and maturation. Beyond directly targeting viral processes, these inhibitors can also restore host immune responses by preserving the nuclear translocation of critical antiviral factors, such as STAT1 and IRF3, which are often inhibited by viral proteins. This multifaceted approach highlights the therapeutic potential of nuclear transport inhibitors in combating viral infections and mitigating their impact on host cellular functions. Host factor inhibitors, such as Ivermectin, offer broad-spectrum antiviral potential by targeting essential nuclear transport receptors like importin-β1 and Crm1. However, their use may be limited due to potential side effects on critical cellular functions. Additionally, the complex clinical spectrum of viral diseases, such as COVID-19, necessitates careful timing and assessment of host-specific inhibitor treatments to maximize efficacy.

In silico drug screening, when integrated with in vitro and in vivo studies, provides an efficient strategy for developing viral-specific nuclear transport inhibitors. Computational methods rapidly identify candidate compounds by screening large chemical libraries for those targeting viral proteins or their interactions with host nuclear transport machinery. These candidates are then validated in vitro to assess their efficacy in disrupting viral nuclear import or export pathways, followed by in vivo studies to evaluate pharmacokinetics, pharmacodynamics, and therapeutic potential. This integrated approach accelerates drug discovery, reducing costs while ensuring specificity and minimal off-target effects. However, viruses may counter these strategies by forming viral condensates, which shield them from antiviral drugs and disrupt host nuclear transport systems, presenting additional challenges for therapeutic intervention [324]. Drugs targeting viral condensates could open other avenues for the development of antiviral therapeutics [97,325]. Coupling viral proteins during nuclear export with the 26S proteasome [67] is another potential strategy to inhibit viral assembly by promoting the degradation of viral proteins [326]. In conclusion, based on our experience with HS-AFM imaging of human NPCs, DNA–protein interactions, viral proteins, viral protein-antibody interactions, and viral protein-organelle interactions, we emphasize the importance of studying nanoscopic real-time interactions between viral components and host nuclear transport factors to inform the design of effective antiviral strategies.

To conclude, viruses have evolved sophisticated mechanisms to exploit the host NPC and nucleocytoplasmic trafficking machinery. By targeting host NTRs and nucleoporins, viruses efficiently gain access to the nucleus, underscoring the critical role of these processes in viral life cycles. This review highlights the complex interplay between viral factors and host determinants, providing valuable insights into the strategies employed by viruses to manipulate nuclear import and export. The therapeutic potential of targeting nucleocytoplasmic trafficking has emerged as a promising avenue for antiviral intervention. Pharmacological inhibitors, which target host nuclear transport systems, impair viral replication and offer significant potential as antiviral strategies. However, the development of such therapies requires continued research to identify and exploit specific vulnerabilities within these pathways, ensuring both efficacy and safety. Advances in high-resolution techniques, such as HS-AFM videography and computational modeling, are revolutionizing our understanding of host-virus interactions at the molecular level. These tools are not only unveiling the intricacies of the viral exploitation of the NPC but also driving the discovery of novel therapeutic targets. By bridging basic research and translational medicine, these cutting-edge approaches are paving the way for innovative antiviral strategies that could mitigate the impact of viral infections. Ultimately, a deeper understanding of viral mechanisms for exploiting nuclear pore transport pathways will enhance our ability to develop targeted therapies, providing a critical foundation for combating existing and emerging viral threats.
